# Diagnosis of bacterial urinary tract infection: Utility of urine myeloperoxidase concentration to predict urine culture results in dogs

**DOI:** 10.1371/journal.pone.0233566

**Published:** 2020-05-22

**Authors:** Jillian Myers Smith, Courtney Thomason, Xiaocun Sun, Elizabeth M. Lennon

**Affiliations:** 1 Department of Small Animal Clinical Sciences, College of Veterinary Medicine, University of Tennessee, Knoxville, Tennessee, United States of America; 2 Office of Information Technology, University of Tennessee, Knoxville, Tennessee, United States of America; University of Lincoln, UNITED KINGDOM

## Abstract

Suspected bacterial urinary tract infections (UTI) are a common cause of overuse and misuse of antimicrobials. A bedside diagnostic test that could accurately predict urine culture results would prevent antimicrobial overuse, but accurate biomarkers have not yet been identified in veterinary medicine. The objective of this study was to evaluate urine myeloperoxidase (uMPO) as a rapidly available, accurate marker to predict urine culture results. We hypothesized that uMPO would be higher in dogs with a positive urine culture than in dogs with a negative urine culture, and that uMPO could be used to aid in the accurate diagnosis of significant bacteriuria. Urine samples were collected from a veterinary university clinical pathology lab. uMPO concentration was measured using a commercially available canine myeloperoxidase (MPO) enzyme-linked immunosorbent assay (ELISA). Following validation, samples from 98 dogs that had a urinalysis and urine culture performed as part of their diagnostic investigation were included. Forty-seven dogs had a negative urine culture and fifty-one dogs had a positive urine culture. uMPO levels were significantly higher in samples that had a positive culture (median 2.13 ng/ml; IQR 0.98–7.07) versus samples that had a negative culture (median 1.07 ng/ml; IQR 0.52–1.84)(p < 0.005). Based on receiver-operator characteristic, a cutoff of 0.55 ng/ml was chosen to maximize sensitivity and specificity. Using a cutoff of 0.55 ng/ml, uMPO had a sensitivity of 70% and specificity of 69% to determine the presence of a positive culture. However, the degree of overlap between groups may preclude the use of this test as a surrogate for urine culture in a clinical setting.

## Introduction

Canine bacterial urinary tract infections (UTIs) are common in general veterinary practice, with up to 14% of dogs affected in their lifetime [1]. However, UTIs can sometimes offer a diagnostic challenge, and inappropriate treatment can lead to antimicrobial overuse. A recent study found that only 51% of dogs presenting to a general practice with clinical signs of UTI who had a urine culture performed as part of their diagnostic workup had a positive urine culture [2]. Furthermore, this same study documented that inappropriate treatment decisions based on urinalysis results led to overtreatment and overuse of antimicrobials in 36% of patients [2]. Challenges in diagnosing UTIs include obtaining a sufficient sample and the cost of urine culture/susceptibility which is considered gold standard for diagnosis but can be expensive [3]. Additionally, culture results are not immediately available, so treatment is often initiated prior to obtaining final culture results. A test that could provide rapid results to corroborate microscopy findings would be beneficial and may prevent unnecessary antimicrobial usage. This is especially important as inappropriate antimicrobial use increased the risk for development of future, resistant UTIs in humans [4]. Therefore, a bedside test that could accurately predict urine culture results would clearly be invaluable in veterinary practice.

A previous study evaluated neutrophil gelatinase-associated lipocalin (NGAL) in dogs with UTI, which concluded that the protein was elevated in infection but was also influenced by the presence of pyuria, which can occur with other diagnoses such as urolithiasis, inflammatory conditions and urinary neoplasia [5]. Increased NGAL could also result from proximal renal tubular epithelial cell damage [5]. In a different study, a point-of-care rapid immunoassay detecting bacterial cell surface antigens in the urine of dogs showed promise, with 97.4% sensitivity and 98.8% specificity [6]. Four of the 200 samples used in this study were collected via free-catch voiding, and 50% of these (2 samples) had false-positive results, indicating that the sensitivity of this test may give false positive results when urine is collected by free catch.

A 2014 study evaluated biomarkers for UTI in humans and concluded that myeloperoxidase (MPO) was significantly elevated in humans with UTI compared to those without UTI [7]. MPO is an enzyme that is found in immune cells, particularly in neutrophils. It contributes to the formation of hypochlorous acid and bacterial destruction. uMPO activity increased in the urine of human patients with UTIs, with 87% sensitivity and 100% specificity for UTI diagnosis [7], and therefore, uMPO may be a promising biomarker for UTI in canines. To the authors’ knowledge, uMPO has not been investigated as a biomarker of urinary tract infection in veterinary patients.

We hypothesized that uMPO would distinguish, with high sensitivity and specificity, between dogs that had significant bacterial growth on urine culture and those with negative cultures. Our objective was to determine if uMPO could be used as a biomarker for significant bacteriuria in dogs.

## Materials and methods

### Animals and sample collection

Urine samples were prospectively collected between February and December 2017 at a single university veterinary teaching hospital. Urine samples were collected from patients as part of their diagnostic workup. Approval for the use of leftover biological laboratory samples from each patient was verified prior to inclusion in this study with written informed owner consent. Samples that had been collected via cystocentesis were brought to the clinical pathology or microbiology laboratory for urinalysis and culture submission, respectively. Samples were included if there was sufficient remaining supernatant from the urinalysis, and if a urine culture was submitted within 24 hours of the urinalysis.

Upon receipt of the samples by the laboratory, the urine was processed according to the university clinical pathology laboratory standard operating procedures as follows: the specific gravity was determined using a refractometer. A dipstick was read using the Clinitek Status instrument, and the urine was centrifuged (370 x g) for 5 minutes. Immediately following centrifugation, the supernatant was collected into a cryovial, and a standard microscopic examination of the urine sediment was performed, and number of red blood cells, white blood cells, and epithelial cells per high power field was quantified. The presence of crystals or other material was reported. The supernatant was placed at -20°C within 1 hour following centrifugation. Samples were then transferred to an ultralow-temperature freezer (-80°C) within 24 hours. For inclusion, a urine culture from a sample obtained via cystocentesis must have been submitted within 24 hours of the urinalysis and refrigerated if not submitted to the laboratory immediately post collection.

Medical records were reviewed and signalment, sex, medication history, and diagnosis were recorded. Samples were considered positive for significant bacteriuria if they had a positive urine culture with ≥1000 colony forming units per milliliter of urine, which is considered to be the standard cutoff of significant growth in a sample obtained by cystocentesis [8]. Samples were considered negative if the urinalysis demonstrated no pyuria (< 5 white blood cells per high power field), no bacteriuria, and a negative culture. This study was designed as an initial inquiry into the feasibility of MPO to diagnose significant bacteriuria, we therefore utilized a tiered approach to assess the utility of uMPO. For Phase 1, we included cases with only a clearly positive or clearly negative urine sample. Cases that had a less clear diagnosis (for example, pyuria with a negative urine culture, urolithiasis, or urinary tract neoplasia) were reserved for a second tier (Phase 2) of testing and future refinement if the test performed well with promising clinical application in Phase 1. Therefore, we reserved cases with the following conditions for future testing: pyuria with a negative urine culture, uncontrolled hyperadrenocorticism, neoplasia of the lower urinary tract system, suspected pyelonephritis, recent immunosuppressive drug therapy (such as treatment with glucocorticoids, mycophenolate, cyclosporine or azathioprine), or history of antimicrobial administration within 2 weeks of culture.

### Laboratory methods

#### Myeloperoxidase ELISA

A commercially available MPO ELISA (MyBioSource Inc., San Diego, CA) was used to determine urine MPO concentrations. The kit has been validated for use in canine serum samples [9], and the manufacturer lists urine as an acceptable sample medium for test use (MyBioSource Canine Myeloperoxidase, product instructions version 09.3.1).

The ELISA was performed as instructed by the manufacturer and absorbance was measured on a Synergy/HTX multi-mode plate reader (Biotek Instruments, Inc. Highland Park, VA). Sample concentrations were determined based on 4 parameter standard logistic curves using analysis of a standard curve. All samples were tested in duplicate. The mean MPO concentration, standard deviation, and coefficient of variation were calculated to determine intra- and inter-assay variability. Recovery was calculated by spiking kit standards into urine samples with a range of pH and specific gravity measurements. Standards at a range of concentrations from 32.5–1000 ng/ml were spiked into urine 4–6 times to determine recovery. Intra-assay and inter-assay coefficients of variation (CV) were calculated by determining the mean and standard deviation (SD) for each sample and then dividing the SD by the mean. Assay precision was assessed by determining intra-assay variability between duplicates of all samples. Inter-assay variability was performed by comparing aliquots of 11 repeated samples between the different assays. Over a period of 3 months, 3 total MPO kits of the same lot number were used, with the 11 samples being repeated between 2 or 3 assays each.

#### Statistical analysis

All final values were normalized by dividing the uMPO concentration by the specific gravity (SG) in the following way: normalized uMPO = uMPO/((SG-1)/1000) as suggested by Williams, et al. [10] for the normalization of urine for biochemical analysis. In humans, specific gravity has been shown to provide superior normalization of metabolites present in the urine compared to normalization to urine creatinine concentration [11–13]. Normalized uMPO data was analyzed using a two-sample t-test to detect the effect of culture status. A non-parametric Kruskal-Wallis test was applied to compare the group means as a diagnostic analysis on residuals that violate the assumption of normality. Backward regression analysis was conducted to select the significant subset of proposed independent variables among age, gender, diabetic status and culture status and a following two-way ANOVA was performed to determine the effects of diabetic status and culture status on normalized uMPO. Normality assumption on residuals was examined using a Shapiro-Wilk test. Rank data transformation was applied as a diagnostic analysis on residuals indicated violation of model assumption of normality. Additional logistic regression analyses were conducted to evaluate the effects of age, sex, or diabetic status and normalized uMPO on culture status and to determine the effect of normalized uMPO on culture status in non-diabetic subjects only. A receiver-operator characteristic curve was derived from the logistic regression to demonstrate sensitivity and specificity of the model. Statistical significance was identified at the level of 0.05. All analyses were conducted in JMP pro 14 for Windows 64x (SAS institute Inc., Cary, NC).

## Results

### Assay validation in urine

Validation of the MPO assay on urine was first performed. For intra-assay variability, coefficients of variation (%CV) ranged from 4.8%– 8.7%. The inter-assay variability was 18.34%. The acceptable range of %CV is typically accepted as <15% for intraassay variability and <20% for interassay variability [14]. Based on urine spiked with standards, average percent recovery was determined to be 45% for this assay. Although the percent recovery was low, precision was high, and the recovery was predictable between multiple samples of different concentration (sample specific gravity ranged between 1.028–1.050) and pH (ranged from pH 6–8.5), and therefore deemed acceptable.

Out of 425 urine samples, samples from 98 dogs met the inclusion criteria and were included in the study. The average age was 8.13 years (range 1–15 years). Five intact females, 4 intact males, 51 spayed females, and 38 neutered males were included. Reasons for exclusion of samples included lack of sufficient remaining urine volume, unknown final diagnosis/incomplete medical record or excluded comorbidity and antimicrobial or steroid administration outside of the accepted timeframe set by inclusion requirements.

Fifty-one samples were positive for bacterial growth, and of these, 8 of these samples had growth of more than one bacterial species in the urine. All sample cultures in this study had bacterial counts of ≥75,000 CFU/mL. Samples with two isolates were counted separately in each category for a total of 59 individual isolates grown. Sixty-six percent (39/59) were positive for *Escherichia coli*, 15% (9/59) were positive for *Enterococcus* spp., 8% (5/59) were positive for beta hemolytic *Streptococcus* spp., 5% (3/59) were positive for *Klebsiella pneumoniae* and <1% each (1/59) were positive for *Enterobacter* sp., *Staphylococcus pseudointermedius* and *Proteus mirabilis*.

### Myeloperoxidase

**Fig 1:**
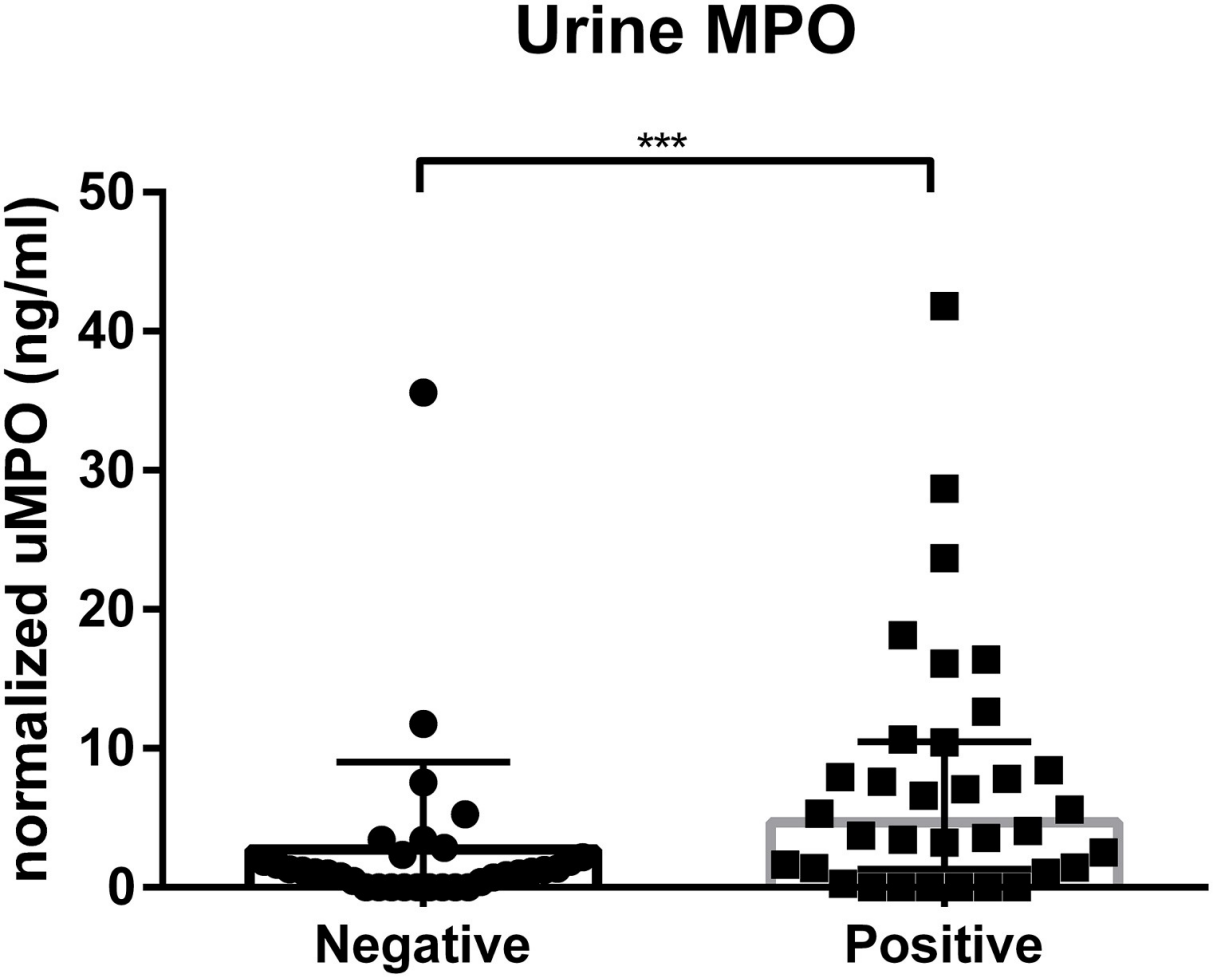
Urine myeloperoxidase concentrations in dogs with a negative or positive urine culture, normalized to specific gravity. Each dot represents an individual patient result. The bar represents the median, and the whiskers represent the interquartile range. *** p < 0.002 uMPO = urinary myeloperoxidase.

**Fig 2:**
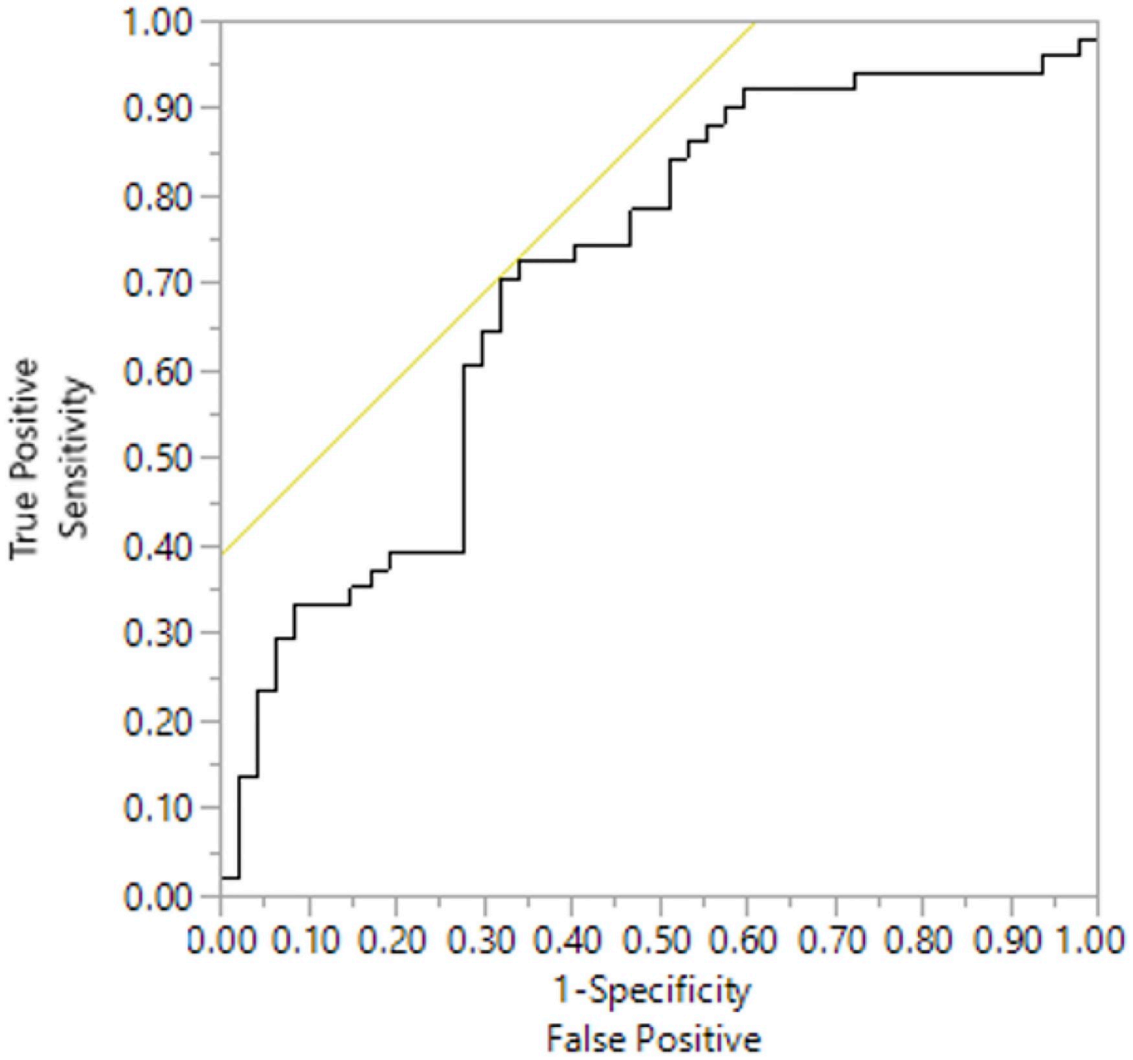
ROC curve analysis for urinary myeloperoxidase. ROC curve analysis revealed that the optimal cutoff for uMPO was 0.55 ng/ml, which resulted in a sensitivity of 70% and specificity of 69% for diagnosis of significant bacteriuria. The yellow line represents the maximum for sensitivity and specificity (equally important). Sensitivity is 100% at cut-off point of 0.27 ng/ml and specificity would be 100% when a cut-off point of 0.98 ng/ml. AUC defined as 0.71. Sensitivity is displayed along the y-axis, and 1-specificity along the x-axis. Where abbreviations are: ROC: receiver operating characteristic curve; AUC: area under the curve; MPO myeloperoxidase.

Forty-seven negative samples (median 1.07 ng/ml, range 0.52–1.84 ng/ml) and 51 positive samples (median 2.13 ng/ml, range 0.98–7.07 ng/ml) were included. Dogs with a positive urine culture had significantly higher uMPO values than dogs without evidence of a UTI and a negative culture (Fig 1). A two-way ANOVA showed significantly lower MPO in negative urine cultures than positive urine cultures (p = 0.001). Receiver operator characteristic (ROC) curve analysis was used to determine an optimal cutoff value to distinguish between dogs with a UTI and those without. The best performance was an uMPO of 0.55 ng/ml to give a sensitivity of 70% and specificity of 69% (Fig 2). uMPO had a positive predictive value of 64.5% and a negative predictive value of 69.4%. Parameter estimates were calculated to examine any significant effect from age (p = 0.65), sex (female (p = 0.55), female spayed (p = 0.40), male (p = 0.77)), and none were found to be significant. Backward regression analysis indicated that only culture and diabetic status have significant effects on uMPO. A two-way ANOVA revealed both diabetic status and culture status have significant effects on uMPO; with non-diabetics having significantly higher uMPO than diabetics (p = 0.03). Even when the diabetic population was removed from the analysis, a logistic regression analysis showed that uMPO remains significantly elevated in positive cultures (p = 0.03).

## Discussion

The results of this study demonstrate that uMPO is significantly increased in dogs with a positive urine culture compared to those with negative cultures. However, given the sensitivity and specificity, the clinical utility as a bedside test in an individual patient is questionable and may not always accurately predict urine culture results. Our results indicate that while there may be appreciable differences between the concentration of uMPO present in urine samples with bacterial growth and those without, in this particular population, it was not enough to differentiate a positive versus a negative sample in an individual patient with confidence, as there was marked overlap in values between these two groups. uMPO had a sensitivity of only 70% and a specificity of 69% to determine if infection is present in a sample, which reiterates the difficulty in discerning between a positive and negative urine sample in the population.

MPO is an enzyme found within the azurophilic granules of neutrophils, and it is present in smaller amounts in monocytes and macrophages. MPO is important in innate immunity and is an integral part of neutrophilic oxidative bactericidal activity [15]. In people, uMPO activity is increased in the urine of patients with a UTI [7] and decreasing levels of uMPO have been suggested as a means to monitor UTI treatment efficacy [16]. In animals, it has been shown that neutrophil MPO concentrations may increase to double the normal level in non-fatal bacterial infections [17]. Thus, using MPO as a marker for infectious diseases, such as a significant bacteriuria and UTIs, could potentially be a useful diagnostic tool.

The results of the present study indicate that uMPO concentrations are increased in dogs with positive urine cultures compared to negative urine cultures. However, values from dogs with positive and negative cultures overlapped. uMPO can be affected by disease conditions other than UTIs and significant bacteriuria, which may have resulted in elevated or decreased concentrations in some dogs with concurrent conditions. The population of dogs presenting to a tertiary referral center will often have many concurrent diseases, and therefore this population of samples had a multitude of comorbidities. We elected to reserve urine samples for a second tier of testing from animals with disease that have shown to affect MPO, relative to the population seen. Because the initial results were not promising, we did not pursue the second tier of testing in dogs with comorbidities known to affect MPO concentration or dogs with pyuria but negative urine culture. Dogs with hyperadrenocorticism were excluded because of the effect endogenous steroids may have at reducing an inflammatory response to urinary infection, and therefore reducing uMPO. Dogs with known lower urinary tract neoplasia were also excluded as neoplastic conditions may increase the level of reactive oxygen species such as MPO [18]. Another disease that can influence MPO levels in humans is diabetes mellitus. Diabetes mellitus has been found to increase MPO levels in some humans [19], whereas other studies suggest that it may falsely lower the level of MPO [20]. Given the variable influence diabetes can have on MPO activity in people, we elected to include diabetics in this study to evaluate for any identifiable influence that diabetic status could have on uMPO concentrations in canines. In the data presented here, urine samples from non-diabetics had significantly higher uMPO values as compared to urine from diabetic patients. This finding supports previous literature in humans, where poor glycemic control is thought to lead to alterations in neutrophil function (abnormal bactericidal activity, phagocytosis and chemotaxis) and enzyme activity, thus causing reduction in MPO production and activity in diabetic patients [21].

Some potential causes for the relatively low sensitivity and specificity of uMPO for diagnosis of significant bacteriuria include lack of specificity as a biomarker. Systemic inflammation can influence MPO concentrations. Local increases in MPO have been demonstrated to increase locally in spinal inflammatory damage in mouse models [22], and intestinal MPO concentrations are increased in dogs with chronic enteropathy [23]. Additionally, in humans with inflammatory liver diseases, increased oxidant production by Kupffer cells from hepatic inflammation can increase levels of MPO locally [18]. Alternatively, MPO can be decreased in widespread infections. ‘Secondary’ MPO deficiency has been correlated with neutrophilic consumptive conditions, such as sepsis, and other overwhelming infections, such as parvovirus, pneumonia, and pancreatic abscesses [24].

A limitation of this study is that urine samples were defined as positive or negative for bacterial growth based on culture results. Although culture aids in the diagnosis of urinary tract infection, we did not consider clinical signs and subclinical bacteriuria in our study design. We chose to categorize samples into positive or negative based on culture results alone. The prevalence of subclinical bacteriuria has been documented in 2–9% of the healthy canine population [25], and this estimate is likely higher in populations with other diseases such as endocrinopathies, obesity, and paralyzed dogs [26]. Subclinical bacteriuria is defined by the International Society for Companion Animal Infectious Disease Guidelines as "the presence of bacteria in urine based on a positive bacterial culture from a properly collected urine specimen in the absence of clinical evidence of infectious urinary tract disease" [26]. It is therefore possible that dogs with subclinical bacteriuria were included in this study based purely on their culture results.

The sensitivity and specificity of uMPO concentrations in our population of dogs likely precludes its clinical use, as there is too much overlap within the data for an accurate prediction of whether significant bacteriuria is present in an individual patient, and we elected not to proceed with Phase 2 of testing. However, re-evaluation of uMPO could be considered in a healthy population without comorbidities. Another sample set of animals presenting only for UTI investigation could be assessed, and these values may indicate that uMPO is, in fact, more reliable without the influence of other diseases. However, in any case, the diagnostic utility of uMPO as a rapid surrogate marker for urine culture to aid in the diagnosis of UTI is likely to be low.

## Conclusion

While uMPO has been documented to be a possible biomarker for determining the presence of UTIs in people, the results of this study indicate that in this canine population this biomarker is unlikely to be useful as a rapidly available surrogate marker until culture results become available.

Our study indicates that while there may be appreciable differences between the concentration of uMPO present in urine samples with significant bacteriuria and those without, in our particular set of samples it was not enough to differentiate positive versus negative cultures because there was marked overlap in uMPO concentrations between the two groups.

An additional study that investigates patients with suspected simple UTI's and no other comorbidities may better support our original hypothesis and perhaps allow for the future development of a bedside test to screen patients prior to conducting a urine culture.

## Supporting information

S1 Dataset Individual patient data of normalized uMPO concentration. (XLSX)
